# Hospital Sunday at the Synagogue

**Published:** 1887-06-25

**Authors:** 


### HOSPITAL SUNDAY AT THE SYNAGOGUE.

The Rev. B. Spiers preached on Saturday last on behalf of the Metropolitan Hospital Fund. Taking his text from Isaiah xxxviii. 16, "O Lord, by these things men live, and in all these is the life of my spirit: so wilt thou strengthen me and make me to live," the preacher proceeded to divide his discourse into three parts: our life for God—for ourselves—and for others.

Under the first heading he advocated the necessity of studying God's word, and in connection with this quoted a passage from the Ethics, "If thou hast studied much the law pride not thyself thereon, since thou wast created for that very purpose." He also dealt with the necessity of good and benevolent actions, and strongly deprecated the treating of life and its great issues as matters of no importance.

Under the second heading he pointed out man's superiority to the world in which he, practically, is the principal being; the king of nature; made in the image of the great God. We are to draw from life all the proper and healthy benefits that it can give us. It is our sacred duty to preserve our health and strength, and thus prolong life in order to serve God properly, and to use all things rightly, as those men whom a Rabbi describes as saying before a meal, "Behold! I am about to eat and drink, in order that I may be strong and healthy to serve the Creator."

Thirdly, we are to live for others. Well said Rabbi Hillel, "If I am not for myself, who is to be for me? if I am for myself only, what am I?" Man is a social being, and has duties towards others. If he lives for himself only he mistakes the true purpose of life. God has wisely ordained that the poor and needy shall not cease from the earth, in order that their more fortunate brethren may assist them; and the sages say, "No man is worthy of being called righteous unless he is good and kind to others." The Talmud relates that a Rabbi passed through a town, exclaiming, "Who is anxiously desirous of buying life?" Instantly crowds thronged round him, wondering and impatient. But he read from the Psalms, "Who is the man that desireth life, and liveth many days that he may see happiness? Keep thy tongue from evil, and thy lips from speaking deceit, depart from evil and do good." Thus we learn that a true Israelite not only must observe laws between himself and God, but also between himself and his fellow-man, and observe and do all things that may benefit in every way those around or those less fortunate than himself.

The rev. gentleman then made a stirring appeal on behalf of the hospitals, with special reference to the present Jubilee commemoration. At this time, he thought, when so much happiness and so many joyful observances were spread over the land, a grand and united effort should be made to help those noble institutions in which science and pity do so much to alleviate pain and to prolong life. In connection with the reference to the Jubilee, the preacher paid a graceful tribute to the many virtues and excellencies of the Queen—applying to her Majesty the words of the inspired writer, "Many daughters have done virtuously, but thou hast excelled them all."

In conclusion, a strong appeal was made to the congregation that each one should do his best to alleviate pain and suffering, mindful of the gracious promise, "Your light shall break forth as the dawn, and your healing shall spring forth speedily, and your righteousness shall go before you, the glory of God shall be your reward; then shall you call, and the Lord shall answer, you shall cry for help, and He shall say, Here I am."

